# Regulation of transcription factors on sexual dimorphism of fig wasps

**DOI:** 10.1038/srep10696

**Published:** 2015-06-02

**Authors:** Bao-Fa Sun, Yong-Xing Li, Ling-Yi Jia, Li-Hua Niu, Robert W. Murphy, Peng Zhang, Shunmin He, Da-Wei Huang

**Affiliations:** 1 Key Laboratory of Zoological Systematics and Evolution, Institute of Zoology, Chinese Academy of Sciences, Beijing 100101, China; 2 College of Plant Protection, Shandong Agricultural University, Tai’an 271018, China; 3 Department of Natural History, Royal Ontario Museum, Toronto, Ontario, Canada

## Abstract

Fig wasps exhibit extreme intraspecific morphological divergence in the wings, compound eyes, antennae, body color, and size. Corresponding to this, behaviors and lifestyles between two sexes are also different: females can emerge from fig and fly to other fig tree to oviposit and pollinate, while males live inside fig for all their lifetime. Genetic regulation may drive these extreme intraspecific morphological and behavioral divergence. Transcription factors (TFs) involved in morphological development and physiological activity may exhibit sex-specific expressions. Herein, we detect 865 TFs by using genomic and transcriptomic data of the fig wasp *Ceratosolen solmsi*. Analyses of transcriptomic data indicated that up-regulated TFs in females show significant enrichment in development of the wing, eye and antenna in all stages, from larva to adult. Meanwhile, TFs related to the development of a variety of organs display sex-specific patterns of expression in the adults and these may contribute significantly to their sexual dimorphism. In addition, up-regulated TFs in adult males exhibit enrichment in genitalia development and circadian rhythm, which correspond with mating and protandry. This finding is consistent with their sex-specific behaviors. In conclusion, our results strongly indicate that TFs play important roles in the sexual dimorphism of fig wasps.

Figs and fig wasps, which form a well-known mutualism system, have been studied by many researchers^1^,^2^,^3^. Figs (Angiospermae, Dicotyledoneae, Urticales, Moraceae) are pollinated by their obligate pollinating fig wasps (Insecta, Hymenoptera, Chalcidoidea), and pollinating fig wasps lay their eggs in the figs^4^,^5^,^6^. Both seeds and offspring of fig wasps develop in the figs until figs reach maturity^2^.

Fig wasps exhibit extreme sexual dimorphism. Whereas females have functional wings, compound eyes, and antennae, most males are apterous, with vestigial eyes, antennae, and tarsi, as expected given their functional adaptations to their lifestyles. In general, male fig wasps live within the fig for their entire lives yet adult females emerge and fly to other fig and pollinate them. Such morphological and behavior divergences provide important opportunities for the study of sexual differentiation^7^. The genetic mechanisms driving these sex-specific differences have remained largely unknown. Considering the differences between male and female fig wasps^7^, their organ development, energy metabolism and physiological demands may also differ. The roles played by transcription factors (TFs) in sexual dimorphism are critical for understanding the system^8^. For one TF may include several domains with different functions in an organism^9^, it is also essential to consider TFs in the domain level.

High-throughput sequencing is an important tool for gene expression study. Herein, we apply RNA-Seq to four different developmental stages of the fig wasp *Ceratosolen solmsi* to explore the relationship between TF-expression and sex-specific traits. The genome of this fig wasp, which pollinates *Ficus hispida*, has been sequenced recently^10^. Herein, we mainly focus on transcriptomes to investigate the genetic drivers responsible for phenotypic and behavioral differences. Our results imply that sexual dimorphism closely associates with sex-specific expressions of TFs. These analyses provide insights into the morphological divergence between male and female fig wasps.

## Results

### TF distributions in three species of Hymenoptera

We employed the total functional genes of *C. solmsi*, *Nansonia vitripennis* and *Apis mellifera* to identify their TFs. Herein, we both employ homologous sequence alignment with known TFs of *Drosophila melanogaster*^11^,^12^ and predict them from the DNA-binding domain (DBD) database (Figure S1). In total, predictions indicate that *C. solmsi* has 865 TFs, *N. vitripennis* 1189 and *A. mellifera* 880. Overall, TFs in *C. solmsi* and *A. mellifera* have similar numbers of TFs. The TFs domains information of *C. solmsi*, *N. vitripennis*, *A. mellifera* and *D. melanogaster* were list in Table 1. Among these TFs, zf-C2H2 exhibits the greatest number of domains among the three hymenopterans. Homeobox domains constitute the second most common class of domain in the hymenopterans, as it does in *D. melanogaster*^13^. In contrast, TFs with rve and RVT domains occur only in *N. vitripennis*. These domains were shown to be necessary for most retrotransposons and retroviruses^14^,^15^. The possible degradation of retrotransposons in genomes of *C. solmsi* and *A. mellifera* may owe to the loss of their rve and RVT domains^10^,^16^.

### Evolution of TFs in fig wasp

**Figure 1:**
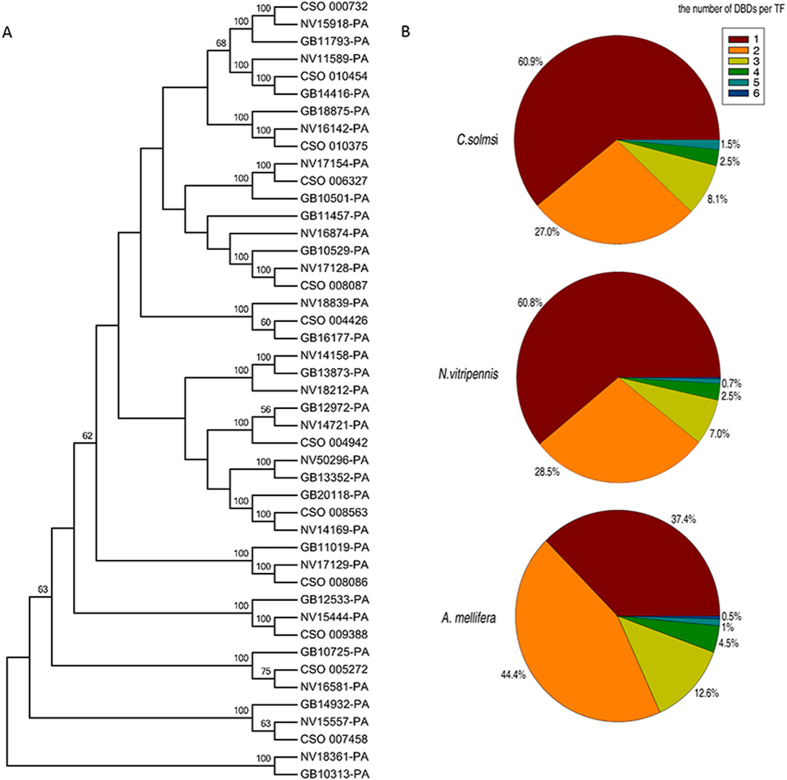
Relationships of transcription factors (TFs) and comparison of DBD numbers per TF in *C. solmsi*, *N. vitripennis* and *A. mellifera*. **A**. ML tree of TFs containing forkhead domains in *C. solmsi*, *N. vitripennis* and *A. mellifera*. Most TFs with forkhead domains show one-to-one correspondence. Number of TFs had forkhead domains: 12: CSO = *C. solmsi*; 16: NV = *N. vitripennis*; and 17: GB = *A. mellifera*. Some TFs such as NV18361-PA *N. vitripennis* with no correspondence in *C. solmsi*. The ML tree is an unrooted tree. Numbers at nodes represent bootstrap values of maximum likelihood. Only bootstrap values above 50 were shown. **B**. The number of DBDs per TF in *C. solmsi*, *N. vitripennis* and *A. mellifera*. The largest proportion of TFs in *C. solmsi* and *N. vitripennis* contain one domain, whereas the largest proportion in *A. mellifera* have 2 DBDs.

Most fig wasp TFs occur in all three species of Hymenoptera. The existence of common TFs indicates an ancient origin and suggests they play vital roles. For example, the phylogeny of forkhead domain containing TFs show that most TFs have a one-to-one relationship among the three hymenopterans (Fig. 1A). Notwithstanding, *C. solmsi* appears to have lost a few TFs with these domains.

The number of DBDs per TF (Fig. 1B) is coincident in *C. solmsi* and *N. vitripennis*. In both species, most TFs contain only one DBD. Further, the number of DBDs per TF relates inversely to the proportion of that class of TF. In contrast, the largest proportion of TFs in *A. mellifera* has 2 DBDs and this accounts for the relatively rich TF-function observed in *A. mellifera*. These findings may reflect the complicated social groups of *A. mellifera*. Moreover, TFs with 5 or more DBDs occur in *N. vitripennis* and *A. mellifera*, but not in *C. solmsi*. Thus, *C. solmsi* possesses a relatively simple composition of TFs in comparison with *N. vitripennis* and *A. mellifera*; this also corresponds with morphological reductions.

### TFs functions related to morphological divergence

**Figure 2:**
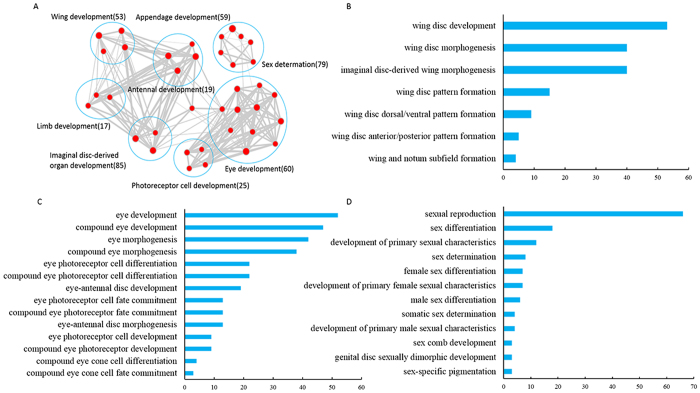
Function of transcription factors (TFs) related to wing, eye development and sexual differentiation of *C. solmsi*. **A**. Enrichment analysis of TFs function. Total TFs were subjected to Gene Ontology (GO) analysis. An enrichment map was constructed by using Cytoscape installed with the Enrichment Map plugin. Red node represents each enriched GO pathway (p < 0.01, FDR q < 0.05, overlap cutoff >0.5). Node size proportional to the total number of genes in each pathway. Edge thickness represents the number of overlapping genes between nodes. GO pathways of similar functions were sorted into one cluster, marked with circles and labels. Gene numbers in each cluster are labeled. **B C** and **D** indicated the number of GO terms classes related to wing, eye development and sexual differentiation, respectively.

To classify TFs functions in the fig wasp, we evaluate the Gene Ontology (GO) term for each TF and look for their functional enrichment. In addition to fundamental biological functions, such as regulation of transcription, gene expression and RNA metabolic process, many TFs of fig wasps associate closely with wing, eye, antenna, limb, appendage and photoreceptor cell development, and other aspects of sexual dimorphism (Fig. 2A, Table S1). Among them, some TFs participate in multiple functions. For example, a few TFs participating in eye development also associate with photoreceptor cell development (Fig. 2A). The 53 TFs associated with wing development assign to 7 related GO terms: wing disc morphogenesis, wing disc pattern formation, wing disc dorsal/ventral pattern formation and so on (Fig. 2B). Similar to wing, a total of 60 TFs participate in eye development and these belong to 14 associated GO terms, such as eye development, compound eye development, eye morphogenesis and compound eye morphogenesis (Fig. 2C). Furthermore, 25 TFs relate to photoreceptor cell development, 19 TFs participate in antenna development and 79 TFs closely associate with sex, such as sexual reproduction, sex differentiation, sexual characteristics and sexually dimorphic development (Fig. 2D). We further examine whether their potential targets connect with respective GO terms, and the results well demonstrate that dimorphic sexually related functions occur in the potential targets of TFs. Similar to TFs, among their potential targets, genes with functions related to wing, eye and other organ development also show enrichment (Figure S2).

### Genes and TFs expression in two sexes across four developmental stages

RNA-Seq was used to investigate the expression of genes and TFs of the sexes in four developmental stages: larva, early pupae (21^st^ day), late pupae (25^th^ day) and adult. Approximately 20 M reads of each sample were mapped to the genome with high quality (Q > 20). Reads were assigned to transcripts based on their overlap with 11412 previously defined reference gene models^10^. Similar to our previous result^10^, most genes were highly expressed (with reads per kb per million mapped reads (RPKM) >10) in the early pupa stage (Figure S3A). Following the development of fig wasps, the number of highly expressed genes reduced in the late pupa and adult stages. Excluding early pupae, only about half of all genes were highly expressed and adult males exhibited the lowest number of expressed genes.

**Figure 3:**
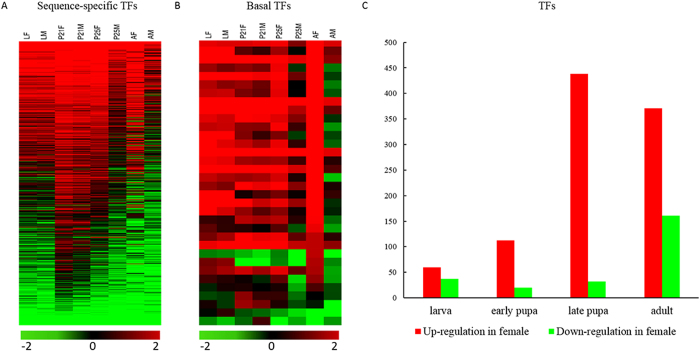
Transcription factor (TF) expression profiles for female and male fig pollinators (*C. solmsi*) at four key life-stages and the number of differentially expressed TFs. ** A** and **B** indicated the expression profiles of sequence-specific TFs (A) and basal TFs (B). LF, larval females; LM, larval males; P21F, early pupal females (21st day after oviposition); P21M, early pupal males (21st day after oviposition); P25F, late pupal stage females (25th day after oviposition); P25M, late pupal stage males (25th day after oviposition); AF, adult females; and AM, adult males. Color gradient illustrated the Z-scores of the gene expression values by calculating as the mean-centered log 2 (RPKM) values divided by the standard deviation for each gene, separately. **C**. Comparisons of significantly up- and down-regulated TF expressions between both genders in the four developmental stages of fig wasps; red columns indicate the number of up-regulated TFs and green columns is the number of down-regulated TFs in females.

TFs cluster into basal and sequence-specific TFs^17^. Basal TFs play a supplementary role in regulating gene expression^18^, while sequence-specific TFs are involved in the regulation of specific target genes^19^. In fig wasps, sequence-specific TFs exhibit similar expression profiles to whole genes (Fig. 3A). The expression profiles of basal TFs consistently show high levels of expression in females at all the four stages, but exhibit low expression (RPKM < 5) in males in the late pupae and adult stages compared to females (*p* < 0.001) (Fig. 3B).

The number of expressed genes differs substantially among the developmental stages based on our conserved criterion that a false discovery rate (FDR)-corrected *p*-value must be less than 0.001 and the fold change greater than 2 (Figure S3B). Larval females have only 612 and 671 genes significantly up- or down-regulated compared to males, respectively. Early pupae females have 1126 and 482 significantly up-or down-regulated genes, respectively. The late pupae and adult stages differentially express a large proportion of genes. In late pupae, 4245 and 465 genes are up- and down-regulated in females, respectively; up-regulated genes are about 10 fold greater than down-regulated ones. This may relate to female-specific pupae development. In adult females, 4044 genes are up-regulated and 1813 are down-regulated significantly. Overall, late pupa and adult females abundantly up-regulate their genes. Intersex patterns of TF expression in the four stages are similar to those of whole genes (Fig. 3C). In the late pupae and adult stages, more than half of the total TFs are differently expressed. Females have more up-regulated TFs than down-regulated ones across all four development stages, especially in the late pupae and adult stages. Adults have the most up-regulated TFs among male developmental stages (Fig. 3C).

### Up-regulated TFs related to female wing, eye and antenna development

**Figure 4:**
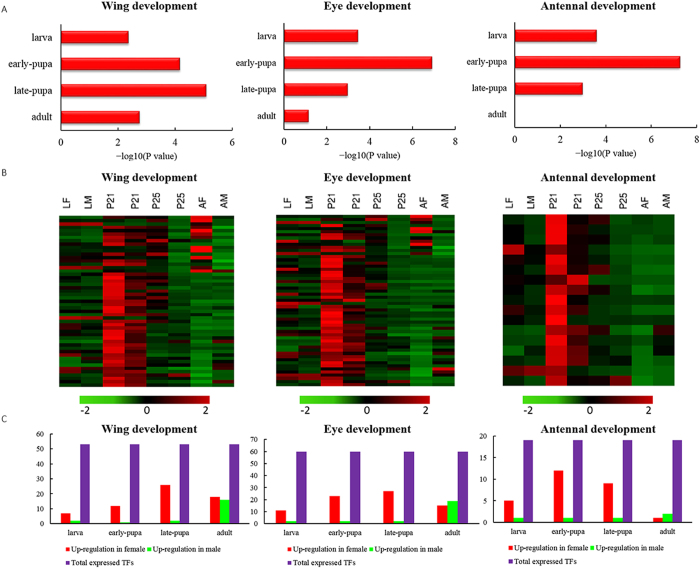
Enrichment of Gene Ontology (GO) terms related to wing, eye and antenna development in up-regulated female transcription factors (TFs) for *C. solmsi*. **A**. GO terms related to wing (left), eye (middle) and antenna (right) development for significantly enriched up-regulated TFs in females in each developmental stage. The X-axis indicates the –log_10_ P value; smaller P values have a greater –log_10_ P value. Column length shows relative significance. **B**. Heatmap plot of TFs related to wing (left), eye (middle) and antenna (right). Color gradient illustrated the Z-scores of the gene expression values by calculating as the mean-centered log 2 (RPKM) values divided by the standard deviation for each gene, separately. **C**. Number of up-regulated TFs related to wing (left), eye (middle) and antenna (right) development between females and males across four development stages.

GO terms serve to identify the functions of enriched TFs in female developmental stages. Further, we explore function enrichment within up- and down-regulated TFs during four development stages in females. In all stages, wing and eye development-related TFs show significant enrichment in up-regulated TFs in females. Thus, these organs may develop from the larva stage, and that development occurs until the adult stage (Fig. 4A). Most of these TFs have the highest levels of expression in early pupae (Fig. 4B). In both sexes, levels of TFs expression decreased in adults compared to other three stages and only a few TFs displayed sex-specific expression (Fig. 4B). The expression patterns of TFs that participate in limb and leg development in females are similar to those of wing and eye (Figure S4).

In contrast, the TFs associated with antenna development differ from those of wing and eye; few TFs have high expression levels in adult females, indicating that antenna development may be completed in the late pupae stage (Fig. 4A). In contrast to females, males do not exhibit functional enrichments of TFs related to antenna development in any stage. To the development stage, pupal females have the greatest number of up-regulated TFs related to wing, eye and antenna development (Fig. 4C).

### Sex-differentially expressed TFs in adults

**Figure 5:**
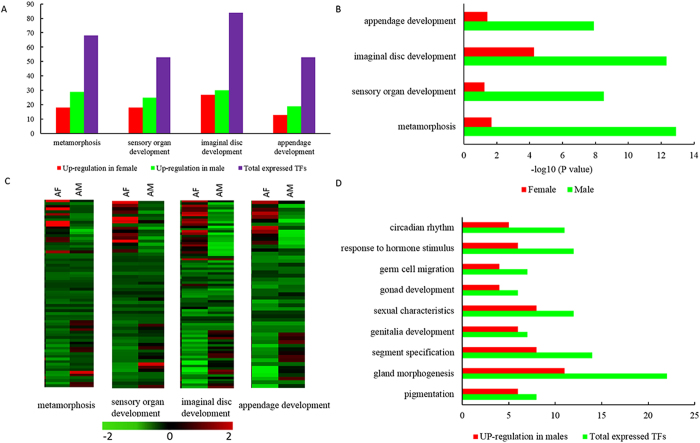
Enrichment of specific Gene Ontology (GO) term in males and adult female *C. solmsi*. **A**. Number of up-regulated transcription factors (TFs) participating in metamorphosis, sensory organ development, imaginal disc development and appendage development between females and males across four development stages. **B**. GO terms participating in metamorphosis, sensory organ development, imaginal disc development, and appendage development in up-regulated TFs between females and males in each developmental stage. **C**. Heatmap plot of TFs related to participation in metamorphosis, sensory organ development, imaginal disc development and appendage development in adults (shown as one part of their expression profile at all four stage, total expression patterns at all four stage are shown in Figure S5). Color gradient illustrated the Z-scores of the gene expression values by calculating as the mean-centered log 2 (RPKM) values divided by the standard deviation for each gene, separately. **D**. Number of up-regulated TFs participating in specific enrichment functions in adult males.

The functional enrichment of up-regulated TFs in male adults indicates that metamorphosis, sensory organ development, imaginal disc development and appendage development have significant enrichments. However, up-regulated TFs in females also have these functional enrichments, but females have fewer than males (Fig. 5A). The enrichment of these functions is significantly higher in males than females yet females have larger total number of up-regulated TFs (Fig. 5B). In either sex, most of these up-regulated TFs mainly involve sex-specific expressions (Fig. 5C). TFs associated with wing, eye, antenna, limb, leg and the development of some other organs display sex-specific high levels of expression in adults and this may contribute significantly to adult sexual dimorphism. Up-regulated TFs in adult males involve enrichment in pigmentation, gland morphogenesis, segment specification, genitalia development, sexual characteristics, gonad development, germ cell migration, response to hormone stimulus, circadian rhythm and other functions. These GO terms do not show significant enrichment in up-regulated TFs of adult females (Fig. 5D). Most TF expression profiles for these functions indicate low levels of expression in females. This corresponds with being male; up-regulated TFs participating in circadian rhythm may relate to protandry. In contrast, female up-regulated TFs associate with eggshell chorion assembly, ovarian follicle cell development, eggshell formation, oogenesis, female gamete generation, reproductive developmental process, and sexual reproduction; these involve female reproduction. Up-regulated TFs in females also have significantly enriched TFs in a variety of biosynthetic processes such as metabolic process and neuron differentiation function, among others. This closely associates with their greater energy requirements and physiological needs.

## Discussion

Accurate and comprehensive predicting of TFs may be critical for understanding sex-specific differentiation. Identified TFs of *D. melanogaster* facilitate the predicting of TFs in *C. solmsi*, *N. vitripennis* and *A. mellifera*. These predictions allow explorations into the evolution of TFs in Hymenoptera, and in particular those of *C. solmsi*. Some TF domain-numbers in *D. melanogaster* differ from those of the hymenopterans. For example, TFs with BESS domains are less prevalent in the Hymenoptera than in *D. melanogaster*. BESS domains in TFs tend to interact with MADF domains. Typical, this interaction occurrs in TF Dip3, which possesses an N-terminal MADF domain and a C-terminal BESS domain. This structure was reported to be conserved in at least 14 proteins of *Drosophila*^20^. Hymenoptera also possess this combination of domains, but the BESS domains with some degradation when compared to *D. melanogaster*. In contrast, TFs with DnaJ and Ank domains are less common in *D. melanogaster* than in the three hymenopterans. This indicates the expansion of TFs with DnaJ and Ank domains. In addition, TFs with PCI domains occur only in Hymenoptera, indicating that they may constitute important adaptations to hymenopteran ecology. For three hymenopteran species, most TFs in fig wasps also occur in other hymenopterans, indicating ancient origins and vital roles these TFs play. In addition, *C. solmsi* possesses fewer TFs and DBDs than do *N. vitripennis* and *A. mellifera*. This may be a consequence of their highly specialized ecology and strict host-specificity. In general, host-specific endosymbionts such as *C. solmsi* show specialized adaptations to life inside their hosts, such as a series of morphological reductions, especially in males. This may relate to reductions in some TF categories. Fig wasps have fewer TFs and greater morphological reductions compared to the other two hymenopterans.

The GO analyses indicated some TFs closely associate with wing, eye, antenna and photoreceptor cell development, sex differentiation. Effectively, all of these TFs likely affect sexual dimorphism. Regarding behavioral divergence of females and males, some TFs participate in biosynthetic process, metabolic process, sensory organ development, neuron differentiation, hormone stimulus and circadian rhythm, which contribute significantly to behavioral divergence.

In all four stages of development, wing- and eye development-related TFs show significant enrichment in up-regulated TFs of females. In addition, TFs that participate in limb, leg development, have patterns of expression that are similar to those of wing and eye. Differing from wing and eye, few TFs associated with antenna development highly expressed in adults; antenna development may reach completion in the late pupae stage. Therefore, sexual dimorphism likely closely associates with up-regulation of TFs in females. A variety of organ-development TFs display sex-specific expression patterns, which may contribute significantly to adult sexual dimorphism. High levels of expression of these TFs in females in early development stage may associate with their relatively complicated morphologies compared with males. Similar expression patterns of TFs and total genes may suggest that these TFs play a critical role in the control of transcriptional regulation. Adult males chew small holes in the walls of galls to enable their mating with females and some males chew holes in the fig wall to enable fertilized females to fly to another fig tree to oviposit and in doing so pollinate figs. Therefore, the adult lives of males and females differ substantially, as do their morphologies^21^. Adult females have a similar numbers of up- and down-regulated TFs and most of these differentially expressed TFs display sex-specific high levels of expression in two sexes, suggesting that these TFs may relate significantly to adult sexual dimorphism, such as TFs related to variety of organ-development. Meanwhile, significant up-regulated or sex-specific expression of TFs also plays an important role in the behavioral divergences of adult fig wasps. Adult males show enrichment in up-regulated TFs involved in pigmentation, gonad development, germ cell migration, response to hormone stimulus, circadian rhythm and other characteristics that associate with male-life and protandry. In contrast, female TFs associated with oogenesis and sexual reproduction are up-regulated significantly, which also relates to their reproduction. Enrichment of female up-regulated TFs occurs in a variety of biosynthetic processes including metabolic process, which closely associates with their energy requirements and physiological needs. Moreover, both females and males have a few TFs with relatively stable levels of expression across all four stages. Excluding functions related to wing and eye development, for some of them also participate in necessary biological processes, such as the regulation of transcription and RNA metabolic process, which may account for their stable expression.

Fig wasps, which have very small bodies, cannot be cultivated ex situ. Their larvae and pupa live inside figs and their sex only can be determined using molecular tools. These attributes make it virtually difficultly to carry out multiple replicates to us, which constrains the strength of our conclusions. Consequently, we use DEGseq^22^, which is suitable for no replicate samples analyses to verify the reliability of the results in the absence of duplicate samples. We use the following strict standards to define differential gene expression: FDR less than 0.001 and fold change greater than 2. Most of candidate gene with differential expression have a fold-change much greater than 2. Thus, qualitative analysis of up- or down-regulation conform to our strict standards. Regardless, herein we provide a new perspective on understanding sexual dimorphism at the level of TFs.

In conclusion, this study provides new insights into the evolution of TFs in fig wasps and sexual dimorphism closely associates with TFs. Further experimentation on these TFs and their potential targets will lead to a more complete understanding of the functional roles TFs play in determining the remarkable sexual dimorphism of fig wasps.

## Methods

### TF prediction in *C. solmsi*

TFs were divided into basal TFs and sequence-specific TFs^17^. Basal TFs played a supplementary roles in regulating gene expression^18^ while sequence-specific TFs were shown to be involved in the regulation of specific target genes^19^. Hence, the methods of prediction differed between the two types of TFs.

Predictions for basal TF were made based on conserved domains. First, basal TFs of *D. melanogaster* were used to find the basal TF domains by employing Pfam^23^. Next, all fig wasp gene sequences were used to predict genes based on fruit fly basal TF domains. In total, 35 fig wasp genes were identified as being basal TFs.

The DBD database (http://www.transcriptionfactor.org) was used to predict sequence-specific TFs and the number of predicted transcription factor repertoires in the DBD database increased from 150 in the initial version to over 700 at present^24^,^25^,^26^. Because this coverage was not sufficiently comprehensive, we also carried out homologous sequence alignment against TFs of *D. melanogaster*. In our analysis, each protein sequence in fig wasp was compared with TFs of *D. melanogaster* in the local database using BLASTP^27^. The results were filtered based on an e-value threshold of 1e^−10^ and a continuous overlap threshold of 33%. Next, these candidate TFs in fig wasp were further confirmed by blasting TFs of *D. melanogaster* back to the fig wasp proteins with the same threshold to confirm reciprocal best hits. Only sequences with reciprocal best hits in fig wasp against TFs of *D. melanogaster* with significant E-value were considered as its transcription factors. Then, for all the remained candidates TF in fig wasp, we searched them against the NR database using BLAST to confirm their TF functions and domains. Thus, we predicted TFs by combining two methods (Figure S1). The DBD-based method predicted a total of 573 sequences as being TFs. After manually removing 40 that were unlikely to be TFs (e.g., enzymes, transmembrane proteins)^26^, we obtained 533 TFs, including 168 new TFs. Using homologous sequence alignments with TFs of *D. melanogaster*^28^, we obtained 697 conserved fig wasp TFs. Combined, the two methods obtained 865 fig wasp TFs. The same approach was used to obtain 1189 TFs in *N. vitripennis* and 880 in *A. mellifera*.

### Evolutionary analyses

To summarize relationships of the TFs in different species, multiple alignment was performed using CLUSTALW2^29^. The raw alignment was corrected manually, and unreliably aligned regions containing gap positions were deleted using GBLOCKS^30^. Maximum likelihood trees were constructed using Phyml^31^ with the best-fit evolutionary model suggested by Prottest 3.0^32^. Branch support values were gained by performing 1,000 bootstraps.

### RNA extraction and sequencing

The transcriptomic data employed herein included eight samples obtained from males and females at four developmental stages: larval (16 days after oviposition); pupal 21 (21 days after oviposition, which is the first day of the pupal stage); pupal 25 (25 days after oviposition, which is the first day of the yellow pupal stage); and adult (30 days after oviposition. Most wasps exit the galls as adults, although they do not emerge from the fig)^10^. Total RNA was isolated using the RNeasy® Micro Kit (Qiagen, Shanghai, China) and treated with DNase (Qiagen, Shanghai, China). A NanoDrop ND-1000 Spectrophotometer (Nano-Drop Technologies, Wilmington, DE, USA) was used to confirm adequate RNA concentration and A260/A280 ratio. RNA was dissolved in 20 μL RNase-free water and kept at −80 °C. Larval females and males that had no distinct morphological divergence were discriminated by the variable splicing pattern of the sex determination gene doublesex^33^. The procedure used 50 ng dissolved RNA of larva fig wasp to synthesize firststrand cDNA by priming with oligo(dT) with TransScript®II First-Strand cDNA Synthesis SuperMix (TransGen Biotech, Beijing, China). The sex of larva individual was then confirmed by PCR of the male-specific splice isoform of doublesex.

For RNA-seq, beads with oligo(dT) were used to isolate poly(A) mRNA. Fragmentation buffer was then added for cutting mRNA into short fragments, which were used as templates. Random hexamer primers were used to synthesize first-strand cDNA. Second-strand cDNA was synthesized using a mixture of buffer, dNTPs, RNase H, and DNA polymerase I. Short fragments were purified with QiaQuick PCR extraction kits and resolved with EB buffer for end repair and addition of poly(A). Next, the short fragments were connected with sequencing adaptors. For amplification with PCR, we selected suitable fragments as templates based on agarose gel electrophoresis. Finally, the libraries were sequenced using an Illumina HiSeq™ 2000.

### Genome data and RNA-Seq data analyses

The genomes of *N. vitripennis* and *A. mellifera*^16^,^34^ were available. The genome and transcriptomes of *C. solmsi* were recently sequenced^10^. The adaptor sequences on raw RNA-seq reads for each sample were stripped by Cutadapt (http://code.google.com/p/cutadapt/) and low quality bases were removed by Trimmomatic^35^. Reads that were less than 20 nt in length or that contained an ambiguous nucleotide were discarded. The remaining reads were aligned to the *C. solmsi* genome using TopHat2 while allowing for up to two mismatches^36^,^37^. Uniquely mapped reads with a mapping quality larger than or equal to 20 were retained for subsequent analyses. The number of reads mapped to each gene in each RNA-seq sample was counted using the HTSeq python package (http://www-huber.embl.de/users/anders/HTSeq/doc/overview.html) with the ‘union’ overlap resolution mode and -stranded = no. The reads-count for each gene was input for the R-package DEGseq to calculate the significantly differentiated expressed genes (Benjamini-Hochberg false discovery rate (FDR) ≤ 0.001 and fold change >2) with the MARS method^22^. For each sample, RPKM was computed as the number of reads which map per kilobase of exon model per million mapped reads for each gene^38^. Expression profiles were generated with Cluster 3.0^39^ and visualized with Java Treeview^40^. Because global patterns of expression were published previously^10^, herein we considered the dominant roles of TFs played in regulating gene expression while focusing on TF-expression and function enrichments related to sexual dimorphism.

### Gene Ontology Enrichment

The GO term for each TF was retrieved by applying blast2go^41^ (http://www.blast2go.com/). Meanwhile, we also investigated the function of fig wasp TFs predicted targets. To predict the potential targets of fig wasps TFs, considering that TFs and their potential targets in *D. melanogaster* have been studied deeply^42^,^43^,^44^ and lots of database related to them, such as FlyTF^11^, REDfly^45^,OnTheFly^46^ and FlyFactorSurvey^47^ have been provided, so, here, to each TF of fig wasp, we firstly get its homologous gene in *D. melanogaster* with reciprocal blast methods as previous mentioned, then based on previous research results in *D. melanogaster*^42^,^43^,^44^ and comprehensive information by integrating several useful databases to get each TF potential targets of *D. melanogaster*. Then, to each target, we get its homologous gene in fig wasp. Through this procedure, targets of each TF in fig wasp were obtained. Then, their GO term were also retrieved by blast2go^41^.

Gene molecular functional enrichment in different gene subsets was determined using Generic Gene Ontology (GO) Term Finder^48^. GO Term Finder located significant GO terms in a list of genes and each gene product may have been represented by three independent structured and controlled vocabularies: molecular function, biological process and cellular component. GO terms with a *p* value of less than 0.01 were determined to be statistically significant. An enrichment map of each specific genes constructed by Cytoscape 2.8.3 installed with the Enrichment Map plugin and the parameter is as follows: p < 0.005, FDR q < 0.05, overlap cutoff >0.5^49^.

### Availability of supporting data

Transcriptomic data were accessed in the National Center for Biotechnology Information Short Read Archive (www.ncbi.nlm.nih.gov/sra) under the accession no. SRP029703.

## Additional Information

**How to cite this article**: Sun, B.-F. *et al.* Regulation of transcription factors on sexual dimorphism of fig wasps. *Sci. Rep.* **5**, 10696; doi: 10.1038/srep10696 (2015).

## Supplementary Material

Supporting Information

## Figures and Tables

**Table 1:** TF domains information in four species.

**DBD**	***C. solmsi***	***N. vitripennis***	***A. mellifera***	***D. melanogaster***
AAA	7	9	4	1
Ank	32	59	29	1
BESS	2	3	5	20
Bromodomain	6	18	15	4
BTB	31	68	44	21
bZIP_1	6	9	9	7
bZIP_2	7	6	9	11
Chromo	9	8	7	4
DnaJ	18	16	13	1
Ets	8	6	8	8
Forkhead	14	16	16	19
GATA	3	7	7	6
HLH	32	39	46	55
HMG _box	17	21	17	17
homeobox	77	82	85	99
HTH_psq	7	18	7	9
LIM	27	27	30	8
MCM	6	8	8	0
MH1	4	5	4	4
Myb	11	12	15	8
PAX	7	7	8	9
PCI	13	16	14	0
PHD	26	29	26	12
rve	0	24	0	0
RVT	0	28	0	0
SET	12	16	13	4
T-box	8	6	6	9
THAP	4	31	8	4
WD40	28	25	23	2
zf-AD	23	26	26	84
zf-BED	2	12	2	6
zf-C2H2	142	120	123	249
zf-C3HC4	9	10	5	2
zf-C4	13	15	22	22
Total	614	802	654	706

Domains information for TFs of *C. solmsi*, *N. vitripennis*, *A. mellifera* and *D. melanogaster*. Numbers indicate the TFs that correspond to DBDs in each species.

## References

[b1] B W. R. Host specificity of fig wasps (Agaonidae). Evolution 24, 680–691 (1970). 10.1111/j.1558-5646.1970.tb01804.x 28564937

[b2] Anstett M. C., Hossaert-McKey M. & Kjellberg F. Figs and fig pollinators: evolutionary conflicts in a coevoled mutualism. Trends. Ecol. Evol. 12, 94–99 (1997). 21237991 10.1016/s0169-5347(96)10064-1

[b3] Janzen D. H. How to be a Fig. Annu. Rev. Evol. Syst. 10, 13–51 (1979).

[b4] Berg C. C. Classification and distribution of Ficus. Experientia 45, 605–611 (1989).

[b5] Machado C. A., Herre E. A., McCafferty S. & Bermingham E. Molecular phylogenies of fig pollinating and non-pollinating wasps and the implications for the origin and evolution of the fig-fig wasp mutualism. J. Biogeogr. 23, 531–542 (1996).

[b6] Freise J. F. Interactive catalogue of world Chalcidoidea. J Appl Entomol 127, 184–184 (2003).

[b7] Weiblen G. D. How to be a fig wasp. Annu. Rev. Entomol. 47, 299–330 (2002). 11729077 10.1146/annurev.ento.47.091201.145213

[b8] Zheng W. *et al.* Transcriptomic analyses of sexual dimorphism of the zebrafish liver and the effect of sex hormones. PloS one 8, e53562 (2013). 23349717 10.1371/journal.pone.0053562 PMC3547925

[b9] Mitchell P. J. & Tjian R. Transcriptional regulation in mammalian cells by sequence-specific DNA binding proteins. Science 245, 371–378 (1989). 2667136 10.1126/science.2667136

[b10] Xiao J. H. *et al.* Obligate mutualism within a host drives the extreme specialization of a fig wasp genome. Genome Biol. 14, R141 (2013). 24359812 10.1186/gb-2013-14-12-r141 PMC4053974

[b11] Adryan B. & Teichmann S. A. FlyTF: a systematic review of site-specific transcription factors in the fruit fly *Drosophila melanogaster*. Bioinformatics 22, 1532–1533 (2006). 16613907 10.1093/bioinformatics/btl143

[b12] Pfreundt U. *et al.* FlyTF: improved annotation and enhanced functionality of the *Drosophila* transcription factor database. Nucleic Acids Res. 38, D443–447 (2010). 19884132 10.1093/nar/gkp910 PMC2808907

[b13] Adryan B. & Teichmann S. A. The developmental expression dynamics of *Drosophila melanogaster* transcription factors. Genome Biol. 11, R40 (2010). 20384991 10.1186/gb-2010-11-4-r40 PMC2884543

[b14] Weiss R. Viral RNA-dependent DNA polymerase RNA-dependent DNA polymerase in virions of Rous sarcoma virus. Rev. Med. Virol. 8, 3–11 (1998). 10398490 10.1002/(sici)1099-1654(199801/03)8:1<3::aid-rmv218>3.0.co;2-#

[b15] Baltimore D. RNA-dependent DNA polymerase in virions of RNA tumour viruses. Nature 226, 1209–1211 (1970). 4316300 10.1038/2261209a0

[b16] Weinstock G. M. *et al.* Insights into social insects from the genome of the honeybee *Apis mellifera*. Nature 443, 931–949 (2006). 17073008 10.1038/nature05260 PMC2048586

[b17] Latchman D. S. Transcription factors: an overview. Int J Biochem Cell Biol 29, 1305–1312 (1997). 9570129 10.1016/s1357-2725(97)00085-x

[b18] Reese J. C. Basal transcription factors. Curr. Opin. Genet. Dev. 13, 114–118 (2003). 12672487 10.1016/s0959-437x(03)00013-3

[b19] Karin M. Too many transcription factors: positive and negative interactions. New Biol. 2, 126–131 (1990). 2128034

[b20] Bhaskar V. & Courey A. J. The MADF-BESS domain factor Dip3 potentiates synergistic activation by Dorsal and Twist. Gene 299, 173–184 (2002). 12459265 10.1016/s0378-1119(02)01058-2

[b21] Herre E. A., Jandér K. C. & Machado C. A. Evolutionary ecology of figs and their associates: recent progress and outstanding puzzles. Annu. Rev. Ecol. Evol. Syst. 39, 439–58 (2008).

[b22] Wang L., Feng Z., Wang X. & Zhang X. DEGseq: an R package for identifying differentially expressed genes from RNA-seq data. Bioinformatics 26, 136–138 (2010). 19855105 10.1093/bioinformatics/btp612

[b23] Finn R. D. *et al.* The Pfam protein families database. Nucleic Acids Res. 38, D211–222 (2010). 19920124 10.1093/nar/gkp985 PMC2808889

[b24] Kummerfeld S. K. & Teichmann S. A. DBD: a transcription factor prediction database. Nucleic Acids Res. 34, D74–81 (2006). 16381970 10.1093/nar/gkj131 PMC1347493

[b25] Wilson D., Charoensawan V., Kummerfeld S. K. & Teichmann S. A. DBD—taxonomically broad transcription factor predictions: new content and functionality. Nucleic Acids Res. 36, D88–92 (2008). 18073188 10.1093/nar/gkm964 PMC2238844

[b26] Vaquerizas J. M., Teichmann S. A. & Luscombe N. M. How do you find transcription factors? Computational approaches to compile and annotate repertoires of regulators for any genome. Methods Mol. Biol. 786, 3–19 (2012). 21938617 10.1007/978-1-61779-292-2_1

[b27] Altschul S. F. *et al.* Gapped BLAST and PSI-BLAST: a new generation of protein database search programs. Nucleic Acids Res. 25, 3389–3402 (1997). 9254694 10.1093/nar/25.17.3389 PMC146917

[b28] Grbic M. *et al.* The genome of *Tetranychus urticae* reveals herbivorous pest adaptations. Nature 479, 487–492 (2011). 22113690 10.1038/nature10640 PMC4856440

[b29] Thompson J. D., Higgins D. G. & Gibson T. J. CLUSTAL W: improving the sensitivity of progressive multiple sequence alignment through sequence weighting, position-specific gap penalties and weight matrix choice. Nucleic Acids Res. 22, 4673–4680 (1994). 7984417 10.1093/nar/22.22.4673 PMC308517

[b30] Castresana J. Selection of conserved blocks from multiple alignments for their use in phylogenetic analysis. Mol. Biol. Evol. 17, 540–552 (2000). 10742046 10.1093/oxfordjournals.molbev.a026334

[b31] Guindon S. & Gascuel O. A simple, fast, and accurate algorithm to estimate large phylogenies by maximum likelihood. Syst. Biol. 52, 696–704 (2003). 14530136 10.1080/10635150390235520

[b32] Abascal F., Zardoya R. & Posada D. ProtTest: selection of best-fit models of protein evolution. Bioinformatics 21, 2104–2105 (2005). 15647292 10.1093/bioinformatics/bti263

[b33] Verhulst E. C., Beukeboom L. W. & van de Zande L. Maternal control of haplodiploid sex determination in the wasp Nasonia. Science 328, 620–623 (2010). 10.1126/science.1185805 20431014

[b34] Werren J. H. *et al.* Functional and evolutionary insights from the genomes of three parasitoid Nasonia species. Science 327, 343–348 (2010). 20075255 10.1126/science.1178028 PMC2849982

[b35] Lohse M. *et al.* RobiNA: a user-friendly, integrated software solution for RNA-Seq-based transcriptomics. Nucleic Acids Res. 40, W622–627 (2012). 22684630 10.1093/nar/gks540 PMC3394330

[b36] Trapnell C., Pachter L. & Salzberg S. L. TopHat: discovering splice junctions with RNA-Seq. Bioinformatics 25, 1105–1111 (2009). 19289445 10.1093/bioinformatics/btp120 PMC2672628

[b37] Kim D. *et al.* TopHat2: accurate alignment of transcriptomes in the presence of insertions, deletions and gene fusions. Genome Biol. 14, R36 (2013). 23618408 10.1186/gb-2013-14-4-r36 PMC4053844

[b38] Mortazavi A., Williams B. A., Mccue K., Schaeffer L. & Wold B. Mapping and quantifying mammalian transcriptomes by RNA-Seq. Nat. Methods 5, 621–628 (2008). 18516045 10.1038/nmeth.1226 PMC13303166

[b39] de Hoon M. J., Imoto S., Nolan J. & Miyano S. Open source clustering software. Bioinformatics 20, 1453–1454 (2004). 14871861 10.1093/bioinformatics/bth078

[b40] Saldanha A. J. Java Treeview—extensible visualization of microarray data. Bioinformatics 20, 3246–3248 (2004). 15180930 10.1093/bioinformatics/bth349

[b41] Conesa A. *et al.* Blast2GO: a universal tool for annotation, visualization and analysis in functional genomics research. Bioinformatics 21, 3674–3676 (2005). 16081474 10.1093/bioinformatics/bti610

[b42] Rhee D. Y. *et al.* Transcription factor networks in *Drosophila melanogaster*. Cell Rep. 8, 2031–2043 (2014). 25242320 10.1016/j.celrep.2014.08.038 PMC4403667

[b43] Aerts S. *et al.* Robust target gene discovery through transcriptome perturbations and genome-wide enhancer predictions in *Drosophila* uncovers a regulatory basis for sensory specification. PLoS Biol. 8, e1000435 (2010). 20668662 10.1371/journal.pbio.1000435 PMC2910651

[b44] Marbach D. *et al.* Predictive regulatory models in *Drosophila melanogaster* by integrative inference of transcriptional networks. Genome Res. 22, 1334–1349 (2012). 22456606 10.1101/gr.127191.111 PMC3396374

[b45] Gallo S. M. *et al.* REDfly v3.0: toward a comprehensive database of transcriptional regulatory elements in *Drosophila*. Nucleic Acids Res. 39, D118–123 (2011). 20965965 10.1093/nar/gkq999 PMC3013816

[b46] Shazman S., Lee H., Socol Y., Mann R. S. & Honig B. OnTheFly: a database of *Drosophila melanogaster* transcription factors and their binding sites. Nucleic Acids Res. 42, D167–171 (2014). 24271386 10.1093/nar/gkt1165 PMC3965123

[b47] Zhu L. J. *et al.* FlyFactorSurvey: a database of *Drosophila* transcription factor binding specificities determined using the bacterial one-hybrid system. Nucleic Acids Res. 39, D111–117 (2011). 21097781 10.1093/nar/gkq858 PMC3013762

[b48] Boyle E. I. *et al.* GO::TermFinder—open source software for accessing Gene Ontology information and finding significantly enriched Gene Ontology terms associated with a list of genes. Bioinformatics 20, 3710–3715 (2004). 15297299 10.1093/bioinformatics/bth456 PMC3037731

[b49] Shannon P. *et al.* Cytoscape: a software environment for integrated models of biomolecular interaction networks. Genome Res. 13, 2498–2504 (2003). 14597658 10.1101/gr.1239303 PMC403769

