# Supplementary material for: Regulation of transcription factors on sexual dimorphism of fig wasps
Source: Sci Rep. 2015 Jun 2;5:10696. doi: 10.1038/srep10696 (PMC4451555; doi:10.1038/srep10696)
Supplement: Supporting Information [file srep10696-s1.pdf]

Regulation of transcription factors on sexual dimorphism of fig wasps

Bao-Fa Sun[1*], Yong-Xing Li[1,2*], Ling-Yi Jia[1], Li-Hua Niu[2], Robert W. Murphy[3], Peng Zhang[1], Shunmin He[1] & Da-Wei Huang[1,2]

List of additional files:

Additional Figure 1. Flow chart for TF prediction.
Basal transcription factors (TFs) predicted based on the conserved domains of basal TFs from *Drosophila melanogaster*. We combined the common TF-prediction method using the DBD database and homologous sequence alignment with *D. melanogaster*-specific TFs in FlyBase.

Additional Figure 2. Function of transcription factors (TFs) predicted targets related to wing, eye and appendage development of *Ceratosolen solmsi*.
A, B and **C** indicated the number of GO term classification related to wing, eye and appendage development, respectively.

Additional Figure 3. Whole gene expression profiles for female and male fig pollinators (*C. solmsi*) at four key life-stages and the number of differentially expressed genes.
A. Expression profiles of whole genes. Color gradient illustrated the Z-scores of the gene expression values by calculating as the mean-centered log 2 (RPKM) values divided by the standard deviation for each gene, separately. LF, larval females; LM, larval males; P21F, early pupal females (21st day after oviposition); P21M, early pupal males (21st day after oviposition); P25F, late pupal stage females (25th day after oviposition); P25M, late pupal stage males (25th day after oviposition); AF, adult females; and AM, adult males.
B. Comparisons of significantly up- and down-regulated gene expressions between both genders in the four developmental stages; red columns indicate the number of up-regulated genes and green columns show the number of down-regulated genes in females.

Additional Figure 4. Enrichment of Gene Ontology (GO) terms related to limb and leg development in up-regulated female transcription factors (TFs) for *C. solmsi*.
A. GO terms related to limb (left) and leg (right) development for significantly enriched up-regulated TFs in females in each developmental stage. The X-axis indicates the $-\log_{10}$ P value; smaller P values have a greater $-\log_{10}$ P value. Column length shows relative significance.

B. Heatmap plot of TFs related to limb (left) and leg (right). Color gradient illustrated the Z-scores of the gene expression values by calculating as the mean-centered log 2 (RPKM) values divided by the standard deviation for each gene, separately.
C. Number of up-regulated TFs related to limb (left) and leg (right) development between females and males across four development stages.

Additional Figure 5. Heatmap plot of TFs related to participation in metamorphosis (A), sensory organ development (B), imaginal disc development (C) and appendage development (D) in four development stages. Color gradient illustrated the Z-scores of the gene expression values by calculating as the mean-centered log 2 (RPKM) values divided by the standard deviation for each gene, separately.

Figure S1

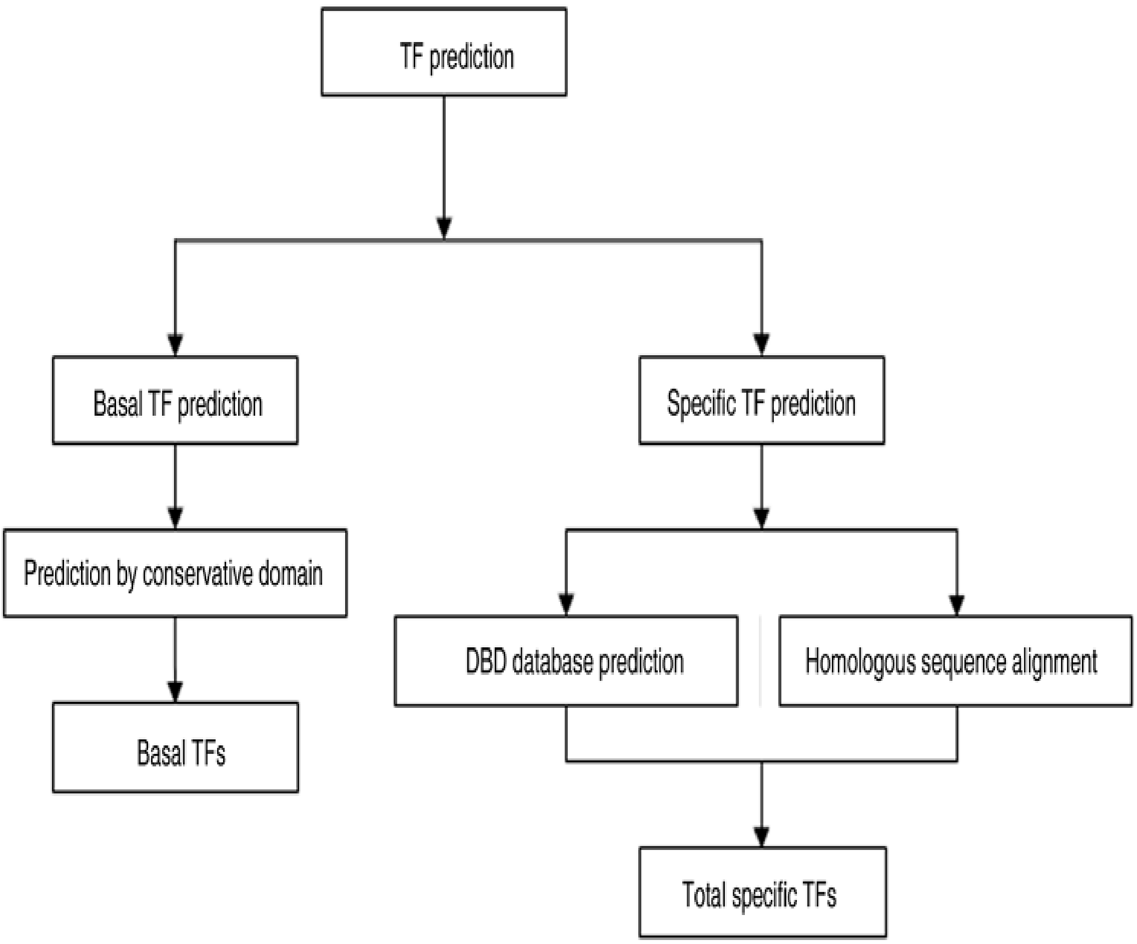

TF prediction
Basal TF prediction
Specific TF prediction
Prediction by conservative domain
DBD database prediction
Homologous sequence alignment
Basal TFs
Total specific TFs

Figure S2

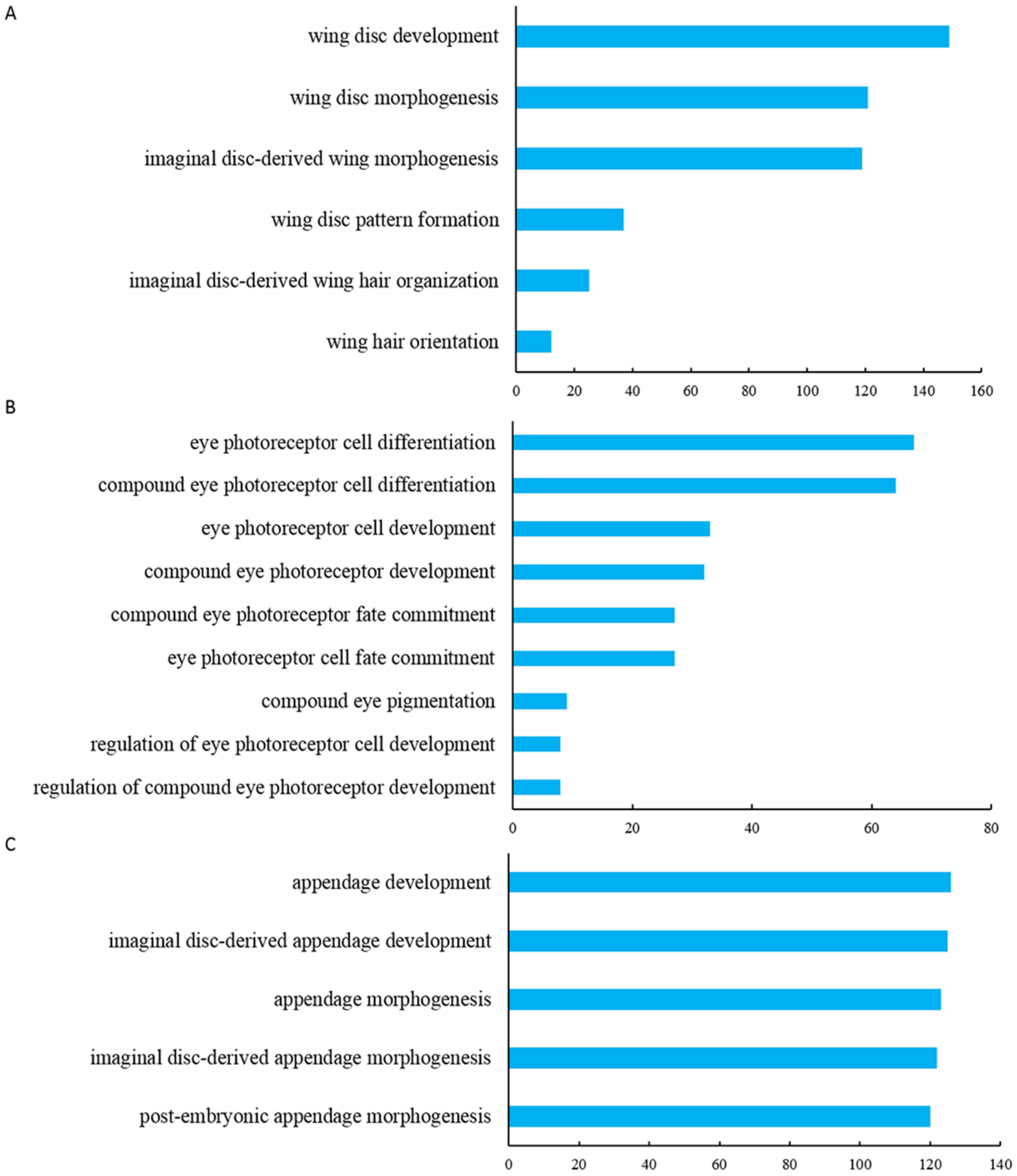
A
wing disc development
wing disc morphogenesis
imaginal disc-derived wing morphogenesis
wing disc pattern formation
imaginal disc-derived wing hair organization
wing hair orientation
0 20 40 60 80 100 120 140 160
B
eye photoreceptor cell differentiation
compound eye photoreceptor cell differentiation
eye photoreceptor cell development
compound eye photoreceptor development
compound eye photoreceptor fate commitment
eye photoreceptor cell fate commitment
compound eye pigmentation
regulation of eye photoreceptor cell development
regulation of compound eye photoreceptor development
0 20 40 60 80
C
appendage development
imaginal disc-derived appendage development
appendage morphogenesis
imaginal disc-derived appendage morphogenesis
post-embryonic appendage morphogenesis
0 20 40 60 80 100 120 140

Figure S3

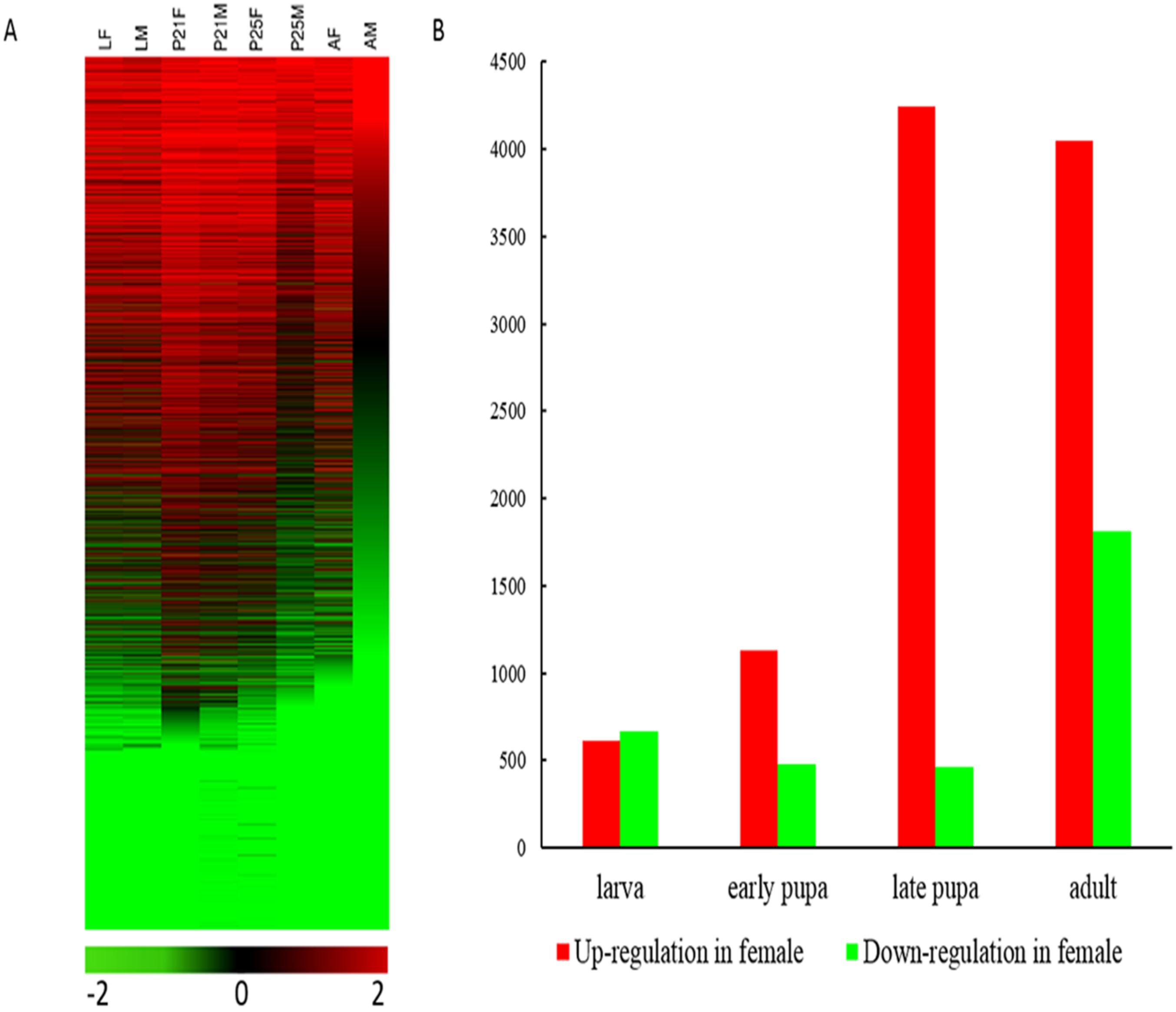
A
LF
LM
P21F
P21M
P25F
P25M
AF
AM
-2
0
2
B
4500
4000
3500
3000
2500
2000
1500
1000
500
0
larva
early pupa
late pupa
adult
Up-regulation in female
Down-regulation in female

Figure S4

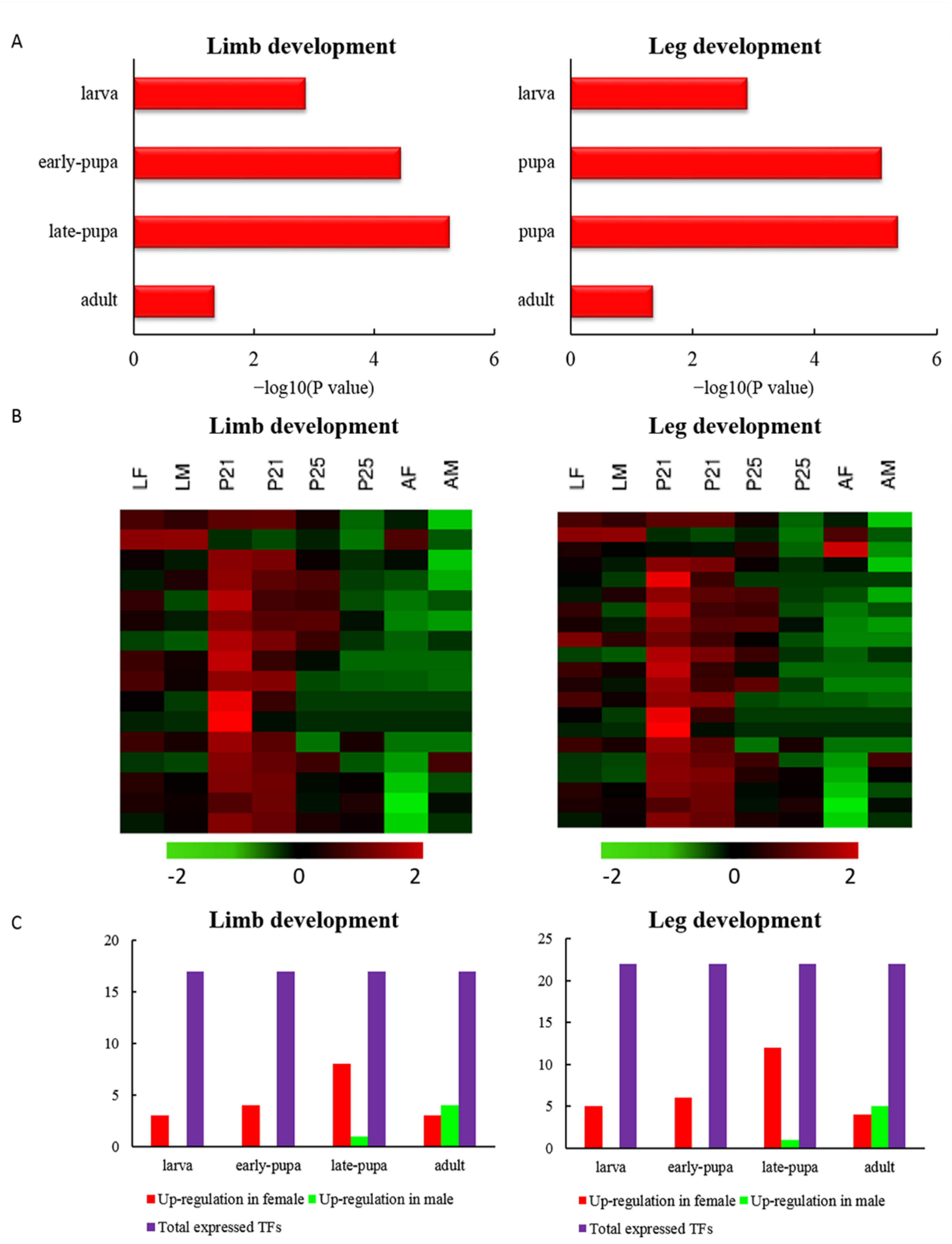
A
Limb development
larva
early-pupa
late-pupa
adult
0
2
4
6
−log10(P value)
Leg development
larva
pupa
pupa
adult
0
2
4
6
−log10(P value)
B
Limb development
LF
LM
P21
P21
P25
P25
AF
AM
-2
0
2
Leg development
LF
LM
P21
P21
P25
P25
AF
AM
-2
0
2
C
Limb development
20
15
10
5
0
larva
early-pupa
late-pupa
adult
Up-regulation in female
Up-regulation in male
Total expressed TFs
Leg development
25
20
15
10
5
0
larva
early-pupa
late-pupa
adult
Up-regulation in female
Up-regulation in male
Total expressed TFs

Figure S5

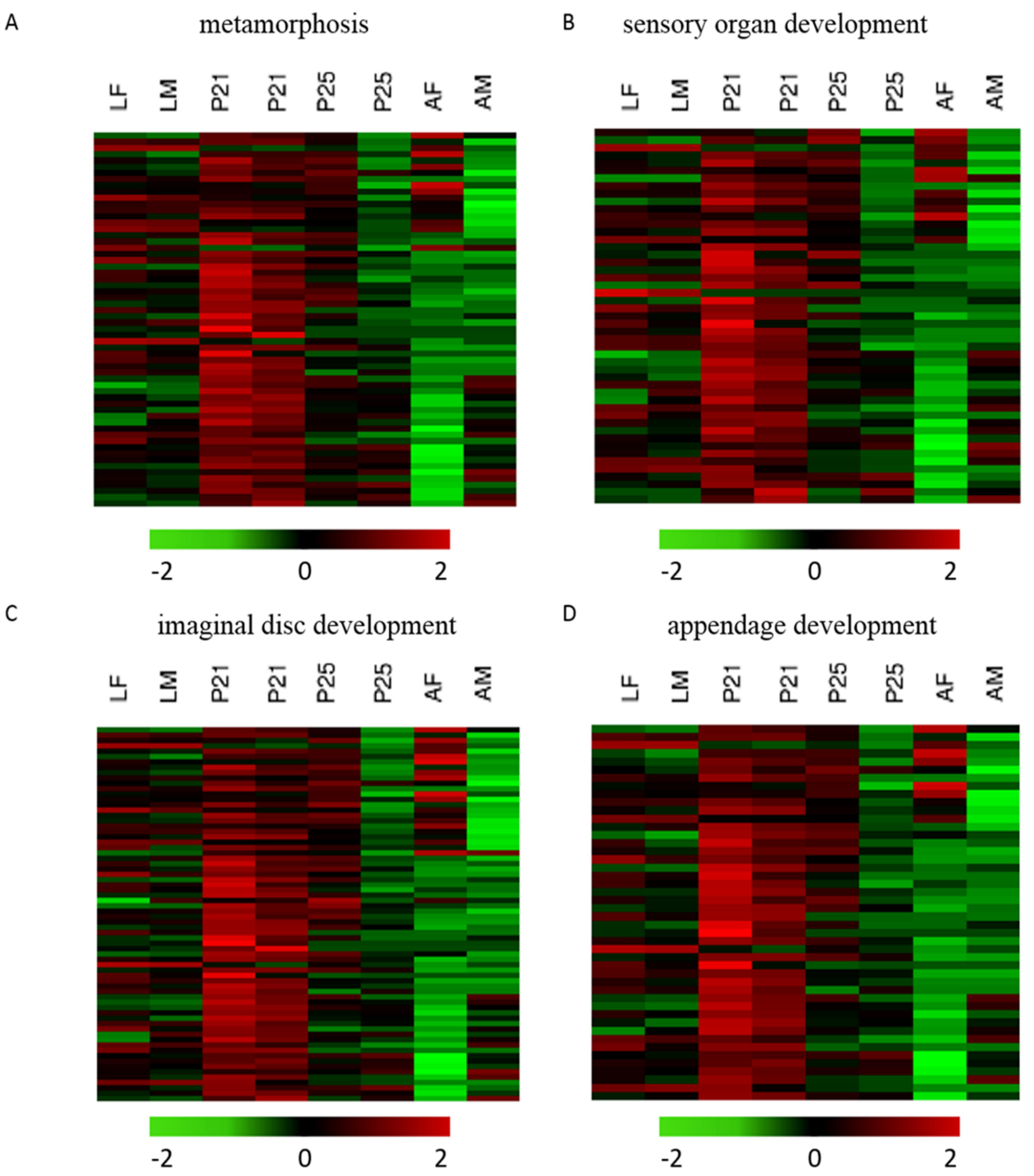

A
metamorphosis
LF
LM
P21
P21
P25
P25
AF
AM
-2
0
2
B
sensory organ development
LF
LM
P21
P21
P25
P25
AF
AM
-2
0
2
C
imaginal disc development
LF
LM
P21
P21
P25
P25
AF
AM
-2
0
2
D
appendage development
LF
LM
P21
P21
P25
P25
AF
AM
-2
0
2
